# Sub-Tenon's bupivacaine injection is superior to placebo for pediatric strabismus surgery: A meta-analysis

**DOI:** 10.3389/fped.2023.1105186

**Published:** 2023-02-16

**Authors:** Zeng Weijuan, Li Zonghuan, Wang Qian, Deng Xizhi, Jiang Bin, Ke Min

**Affiliations:** ^1^ Department of Ophthalmology, Zhongnan Hospital of Wuhan University, Wuhan, China; ^2^ Department of Orthopedics Trauma and Microsurgery, Zhongnan Hospital of Wuhan University, Wuhan, China

**Keywords:** sub-Tenon's injection, bupivacaine, strabismus surgery, meta-analysis, pain

## Abstract

**Background:**

The effect of post-operation sub-Tenon's bupivacaine injection for pediatric strabismus surgery is controversial. The objective of this meta-analysis is to compare the outcome of sub-Tenon injection of bupivacaine and placebo duringstrabismus surgery.

**Methods:**

We searched the databases (Pubmed, Cochrane library and EMBASE) and reference lists systematically. Randomized controlled trials (RCTs) comparing sub-Tenon's bupivacaine and placebo injection for pediatric strabismus surgery were included. The methodological quality was evaluated by the Cochrane risk of bias (ROB) tool. Outcome measurements were pain score, oculocardiac reflex (OCR), additional drug consumption and related complications. RevMan 5.4 was used for the statistical analysis and graph preparation. For the outcomes that are not suitable for statistical analysis, descriptive analysis was performed.

**Results:**

A total of 5 RCTs with 217 patients were finally identified and analyzed. Sub-Tenon's bupivacaine injection showed pain relief within 30 min after operation. But with the extension of time, the analgesic effect gradually disappeared at 1 h. It can reduce the incidence of OCR, vomiting and supplementary drug requirements. However, in terms of nausea, there is no difference between the two groups.

**Conclusion:**

Sub-Tenon's bupivacaine injection can relieve short-term postoperative pain, reduce the incidence of OCR and vomiting, and reduce the use of supplementary drugs in strabismus surgery.

## Introduction

Strabismus surgery is one of the most common ophthalmic operations in children. The cumulative incidence of strabismus at age 7 years was 2.5% in Denmark (1). During the perioperative period, strabismus surgery has an incidence of complications including oculocardiac reflex (OCR), pain and postoperative nausea and vomiting (PNAV).

The OCR is generally defined as a 20% decrease of heart rate or a new arrhythmia during ocular operation. The incidence of OCR during ophthalmic surgery ranges from 32% to 90%, and it is also frequent during strabismus surgery (2). Moreover, the OCR is often accompanied by PNAV, with an incidence of 46%–85% (3, 4). Because opioids and NSAIDs should be used with caution in children, ocular local anesthesia is often adopted as an auxiliary means of postoperative analgesia in pediatric ophthalmic surgery under general anesthesia.

In recent years, sub-Tenon's block is a widely used local anesthesia technique for ocular operations (5). Local anesthetic is injected between the Tenon capsule and sclera to anesthetize the short and long ciliary nerves (6). Children with sub-Tenon's block during retinal and cataract surgeries experience reduced postoperative pain and lower analgesic requirements. It is also reported that this technique can reduce perioperative pain and undesirable side effects during pediatric strabismus surgery (7).

In recent years, several RCTs have compared sub-Tenon's bupivacaine injection with placebo injection for strabismus surgery, but the results are not consistent. Therefore, we conducted this study to evaluate the effect of sub-Tenon's injection of bupivacaine in strabismus surgery.

## Methods

The meta-analysis was conducted in accordance with PRISMA guidelines. The completed PRISMA checklist was uploaded as supplementary files (Supplementary Table S1).

### Including and excluding criteria

The including and excluding criteria was based on PICOS principle (patient, intervention, comparison, outcome and study design). Including criteria: (i) P: pediatric patients diagnosed with strabismus; (ii) I and C: the patients were treated by surgery and sub-Tenon's bupivacaine/placebo injection were performed; (iii) O: at least one outcome (pain score, oculocardiac reflex, additional drug consumption and related complications) was (were) reported; (iv) S: only RCTs were included. No restriction of publication language was set.

The excluding criteria were as the follows: (i): the number of cases less than 10; (ii): the study included both children and adults patients simultaneously and the exact data of pediatric group could not be retrieved.

### Search strategy

Databases including Pubmed, Cochrane library and Embase were systematically searched. RCTs comparing sub-Tenon's bupivacaine injection and placebo injection for pediatric strabismus surgery were included. Medical Subject Headings together with the free words/terms (“sub-Tenon's”, “subtenon's”, “bupivacaine”, “squint” and “strabismus”) were used. The reference lists were also screened and checked for additional studies. The time range of literature retrieval is from the establishment of the database to March 1, 2022.

### Data extraction

The titles and abstracts were screened successively. The unrelated papers were deleted according to the including criteria. The full text of the remaining studies was obtained and checked rigorously. The two authors independently screened the literatures according to the inclusion and exclusion criteria. When there was divergence, the supervisor evaluated and made the decision.

Data were extracted from the included studies, including general information, such as first author, year of publication, country, sample size, age range of included patients, gender ratio, intervention and control measures, study design, etc. At the same time, the outcomes of the literature results were included. If the relevant outcome did not provide specific values, we calculated it from other provided relevant data. Otherwise, only descriptive analysis was made.

### Methodological assessment

The two authors evaluated the quality of the included studies independently, and negotiated or consulted with the superior director to evaluate the divergence. The methodological assessment of included studies was carried out through the risk of bias (ROB) tool provided by the Cochrane Collaboration. The tool consists of seven items including random sequence generation, allocation concealment, blinding of participants and personnel, blinding of outcome assessment, incomplete outcome data, selective reporting and other bias. Each item can be judged as high risk, low risk and unclear risk according to the specific description in the included literature. If there are too many high-risk items or unclear items in the included studies, they will be regarded as low-quality studies and will be excluded or sensitivity analysis will be conducted.

### Statistical analysis

RevMan 5.4 software was used for statistical analysis (8). RR and SMD were respectively used for statistical analysis of dichotomous data and continuous variable, both with 95% confidence interval (CI). The heterogeneity among the included studies was evaluated by *I*^2^. When *I*^2^ > 50%, the heterogeneity was considered to be obvious and the random effect model was selected for analysis. Otherwise, the fixed effect model was adopted. *P* < 0.05 was considered statistically significant.

## Results

### Identification of relevant literature

**Figure 1:**
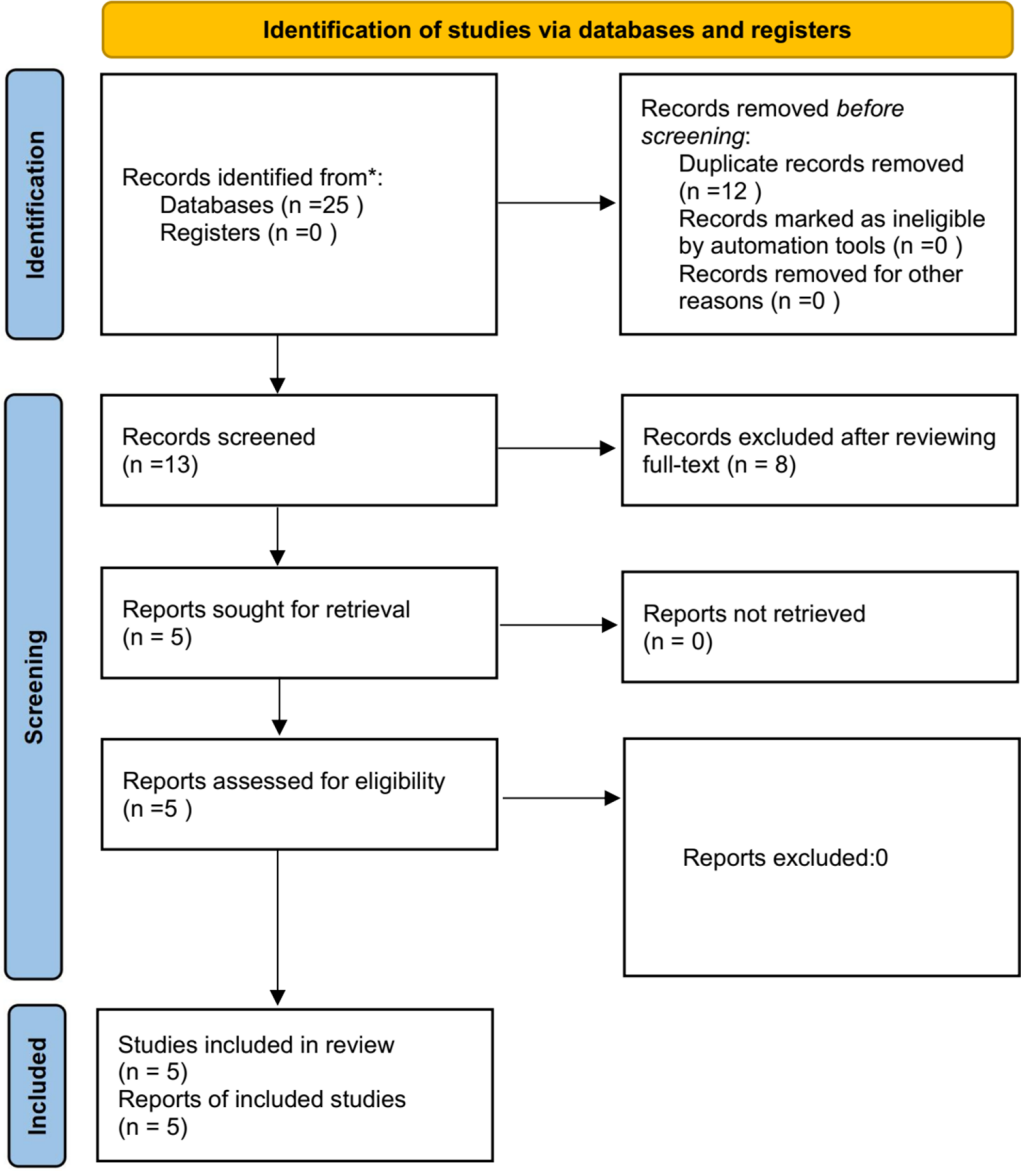
PRISMA flow chart.

The PRISMA flow chart was showed in Figure 1. A total of 25 studies were retrieved from database search and reference list check. Thirteen studies remained after the exclusion of 12 reduplicative studies. Seven studies were excluded after reviewing the title, abstract and full-text. Finally, a total of five RCTs (4, 7, 9–11) with 217 patients were included in this study.

The general information of all included studies was showed in Table 1. The included studies were published between 2005 and 2017. All the studies came from different countries and regions, followed by Iran, Turkey, Egypt, the United States and France. The number of included cases was 33–56, all of which were more than 10. All the cases in the study were minors under the age of 17, including 100 males and 117 females. All pediatric patients received general anesthesia. Sub-Tenon's bupivacaine injection was performed in all the study groups, and placebo or no treatment was used in the control group. Surgery was started 5 min after the sub-Tenon's injection in four studies (4, 7, 9, 10), while sub-Tenon's anesthetic was performed at the end of surgery in one (11). All included studies were RCTs.

**Table 1:** General characteristics of included studies.

Included study	Location	Cases	Age (years)	Sex (M/F)	Intervention	Comparison	Study design	Outcomes
Talebnejad 2017	Iran	50	12 (8–17)	24/26	Sub–Tenon's bupivacaine injection (0.1 ml, 0.5%)	Normal saline injection (0.1 ml)	RCT^†^	OCR^‡^, VAS^§^
Tuzcu 2015	Turkey	40	10 (5–16)	18/22	Sub-Tenon's bupivacaine injection (0.08 ml/kg, 5%)	No treatment	RCT	OCR, Postoperative verbal rating scale, vomiting, nausea
BakR 2015	Egypt	56	3.3 (2–6)	23/33	Sub-Tenon's bupivacaine injection (0.5%, less than 2.5 mg/kg)	Placebo saline injection	RCT	OCR, pain score, nausea, vomiting
Enyedi 2017	USA	33	4.8 (1–7)	19/14	Sub-Tenon's bupivacaine (0.5 ml, 0.75%)	Balanced salt solution (0.5 ml)	RCT	CHEOPS^¶^
Steib 2005	France	38	4.4 (2.5–6)	16/22	Sub-Tenon's bupivacaine injection	Placebo saline injection	RCT	CHEOPS, OCR, PONV

^†^ RCT, randomized controlled trial.

^‡^ OCR, oculocardiac reflex.

^§^ VAS, Visual Analog Scale.

^¶^ CHEOPS, Children's Hospital of Eastern Ontario Pain Score.

### Methodological assessment

**Figure 2:**
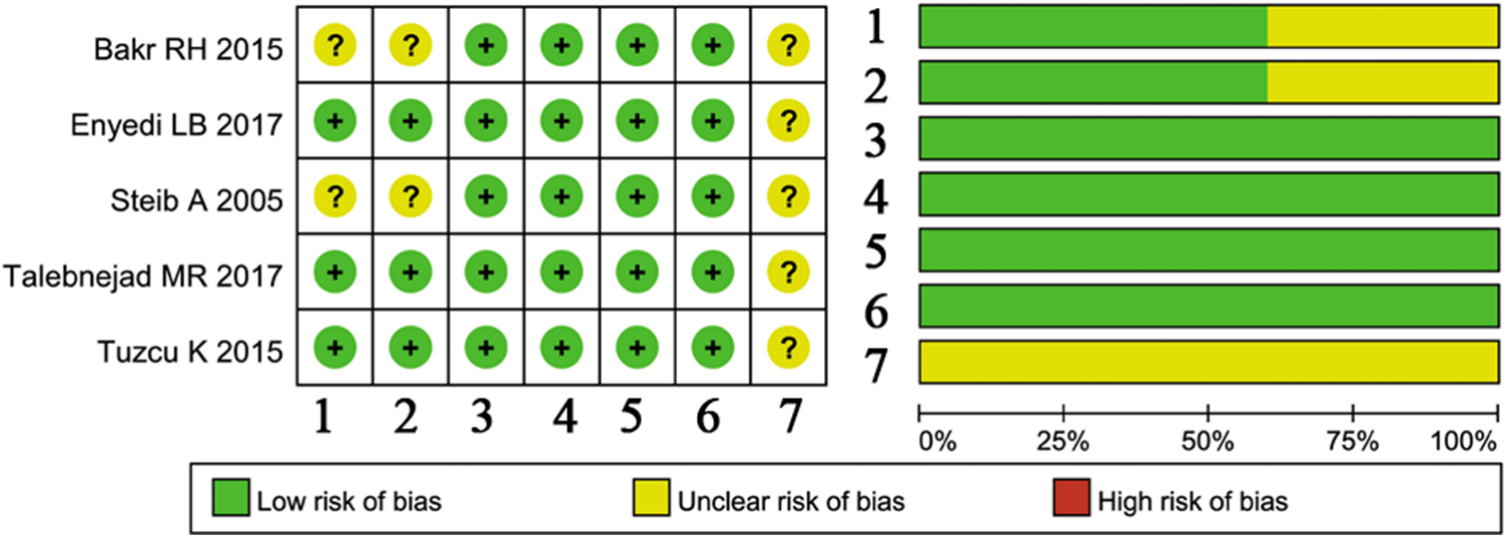
Methodological assessment of included studies by the ROB tool. 1, Random sequence generation; 2, allocation concealment; 3, blinding of participants and personnel; 4, blinding of outcome assessment; 5, incomplete outcome data; 6, selective reporting; 7, other bias.

The methodological quality of included studies was assessed by the ROB tool. As showed in Figure 2, the selection bias, including randomized sequence generation and allocation concealment, was reported and was of low risk in three studies. The blind method for the participants and staff was reported in all studies. Incomplete outcome data and selective reporting were of low risk. The rest items were unclear.

### Outcome measurements

**Figure 3:**
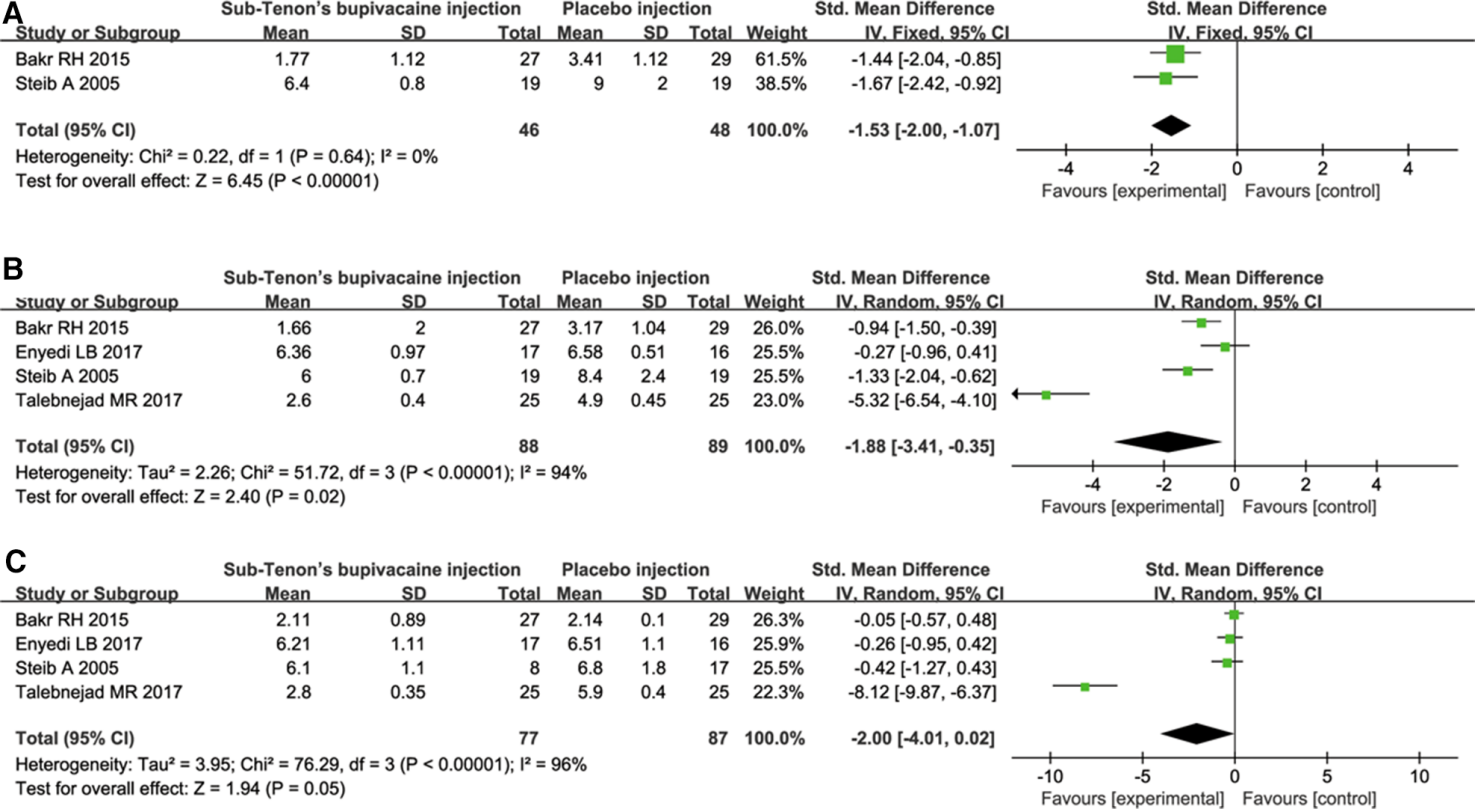
Pain evaluation comparison immediately (**A**), 30 min (**B**) and 60 min (**C**) after the operation.

Pain evaluation was reported in five studies. Tuzcu (4) used postoperative verbal rating scale for pain assessment, and no specific value was given. Thus, data from other four studies were extracted and analyzed. The results showed that, compared with placebo, sub-Tenon's bupivacaine injection showed improvement of pain symptom immediately (Figure 3A), half an hour (Figure 3B) after the operation. However, there was no significant difference in pain score between the two groups 60 min after the operation (Figure 3C).

**Figure 4:**
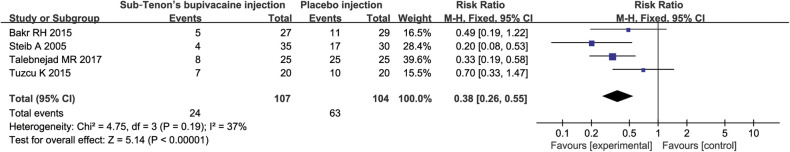
OCR occurrence comparison.

OCR occurrence was recorded in four studies (4, 7, 9, 10) with a total of 211 patients. The incidence of OCR in bupivacaine group was much lower than that in the placebo group (Figure 4).

**Figure 5:**
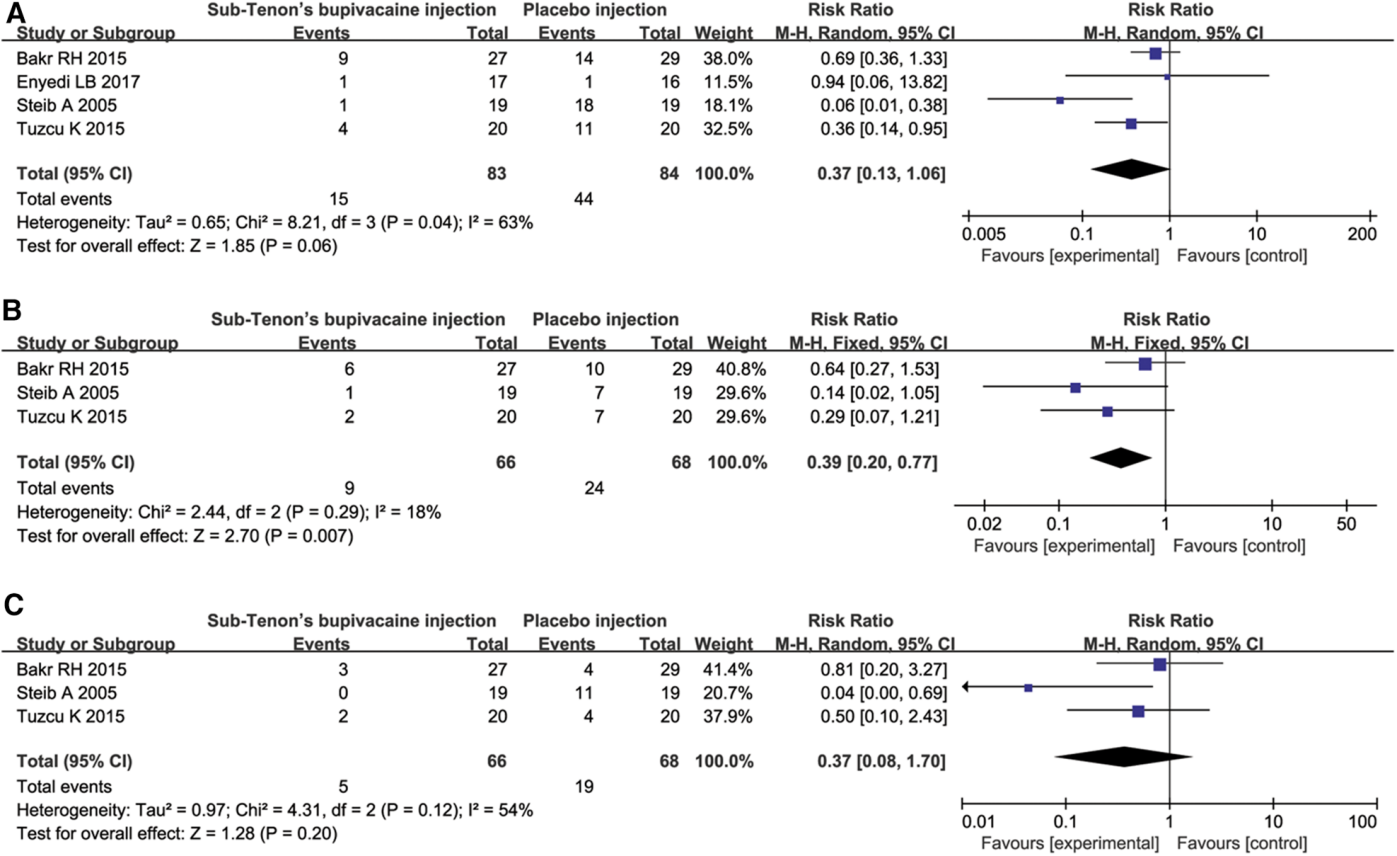
Incidence of PONV (**A**), vomiting (**B**) and nausea (**C**) respectively.

PONV was reported in four studies (4, 7, 9, 10). However, one study (11) did not report nausea and vomiting separately. Therefore, we conducted a pooled analysis of PONV, and then excluded the study and analyzed nausea and vomiting separately. The results showed a tendency of lower incidence of PONV in bupivacaine group (*P* = 0.06, Figure 5A). When analyzed separately, the incidence of vomiting was higher in the placebo group (*P* = 0.007, Figure 5B). There was no significant difference in the incidence of nausea between the two groups (*P* = 0.20, Figure 5C).

**Figure 6:**
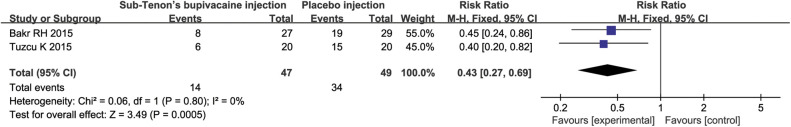
Additional drug requirement.

Two studies (4, 9) reported additional drug requirement. The number of patients who needed additional drugs in the bupivacaine group was significantly less than that in the placebo group (*P* = 0.0005, Figure 6).

Length stay recovery room was reported in two studies, but the timing of the reports was different. The average stay time in the recovery room of s bupivacaine group and placebo group was 95 and 145 min respectively (7). In the sub-Tenon's bupivacaine injection group, 8 of 19 cases were in the recovery room 1 h after operation, and only 3 cases at 1.5 h. In the placebo group, 15 of 19 patients were still in the recovery room for 1.5 h (7). The study by Bakr (9) reported similar results. Two hours after operation, 22 out of 29 children in the placebo group were still in the recovery room, while only 4 of the 27 children in the bupivacaine group.

Other outcomes, including chemosis (7) and heart rate deceleration (10), were reported in one study respectively. Although meta-analysis could not be carried out, the results are more favorable to the bupivacaine group.

## Discussion

Adjuvant local anesthesia has been proved to reduce postoperative pain and the incidence of OCR in pediatric ophthalmic surgery (12). The role of sub-Tenon's bupivacaine injection in cataract surgery has been confirmed (13), but its role in children strabismus surgery is still controversial. Therefore, a total of 5 RCTs and 217 patients were included and analyzed. The results showed that sub-Tenon's bupivacaine injection could reduce the short-term postoperative pain, and the analgesic effect gradually weakened with the extension of time. Meanwhile, it could reduce the incidence of OCR and vomiting, and it also had a certain effect on additional drug requirement and rapid recovery.

In this meta-analysis, all included studies were double-blind RCTs. This kind of rigorous and prospective study design can reduce the bias caused by subjective judgment and other factors, reduce the heterogeneity, and improve the credibility of the conclusion. All the studies were from different countries and regions, which minimized the bias caused by different ethnic and medical conditions.

Regional anesthesia has been proved to reduce postoperative pain and the use of postoperative analgesics. Sub-Tenon's bupivacaine injection under direct visualization is a relatively safe method of local anesthesia (4, 14), As a long-acting local anesthetic, bupivacaine can provide relatively long-term (3–3.5 h) analgesic effect (12). However, in this study, the analgesic effect was only maintained until 30 min after operation, and there was no significant difference in pain score between the two groups at 1 h after operation. On the one hand, the conclusion is more conservative because of the heterogeneity between studies, and on the other hand, it may be related to the time and dose of administration.

OCR refers to a 20% decrease in heart rate or new arrhythmia during eye muscle traction, which occurs in 90% of strabismus surgery (12). Several methods have been proposed to minimized the incidence of OCR. However, the effect of all these methods was limited. Sub-Tenon's bupivacaine injection was confirmed to be effective in reducing the incidence of OCR and PONV (7, 9, 10). However, another study by Tuzcu et al. (4) found that the incidence of OCR and PONV were less commonly for patients with sub-Tenon's bupivacaine injection, but the difference was not statistically significant. In this study, the incidence of OCR and PONV was lower in the experimental group, but there was no significant difference in PONV. When analyzing the data, we found that there was non-negligible heterogeneity of the data in the literature. Some studies only counted OCR, and some mentioned nausea/vomiting. Therefore, in order to make the study more objective and not omit important complications, we conducted statistical analysis on OCR and nausea/vomiting respectively. Interestingly, when the incidence of nausea and vomiting were analyzed separately, the incidence of vomiting in the bupivacaine group was lower, and the difference was statistically significant. Therefore, we speculate that the reason for no significant difference in PONV may be the high heterogeneity (*I*^2 ^= 63%) among the included studies.

Two articles (4, 9) reported the data of additional systemic analgesia requirements, however, both articles did not report which specific drugs were used. In order to minimize the possible bias of PONV caused by opioids use, we excluded both articles in the analysis of PONV. The data of PONV in the remaining two articles (7, 11) were highly heterogeneous, and there was no significant difference using the random effect model. This suggests that the additional use of anesthetic drug may affect PONV, but it does not change the trend of this outcome. The concentration and volume of bupivacaine used in included studies were various. 0.5% bupivacaine is the most commonly used, and the volume varies from 0.1 to 3 ml. This difference is related to the different habits of deach surgeon. It needs further study to determine which concentration and volume are more appropriate.

The concentration and volume of bupivacaine used in included studies were various. 0.5% bupivacaine was the most commonly used, and the volume varied from 0.1 to 3 ml. This difference is related to the different habits of different surgeons. Which concentration and volume are more appropriate needs further study.

The sub-Tenon's bupivacaine injection has few major adverse reactions when applied to children's strabismus surgery. But there have been some sporadic reports of extraocular muscle injury, hemorrhage and globe perforation (15, 16). For children, other minor complications such as conjunctival hemorrhage, petechiae and chemosis may be more common. However, among all 217 patients included in this study, only one patient with chemosis was reported and no major complication was reported. It indicated that the technique is safe for strabismus surgery in children.

This is a secondary analysis based on published data. There are some shortcomings that should not be ignored. Firstly, Although the studies included are well-designed RCTs, there are still some heterogeneities between the studies, such as the age difference of the included patients, the choice of different pain scales and the different dosage of bupivacaine injection, which may have certain impacts on the final results. For strabismus surgery, the degree of pain and the incidence of OCR may be different with different surgical methods. However, several included literatures did not report the details of surgery, and the methodology focused on the description of anesthesia. Therefore, the clinical heterogeneity among the included studies should not be ignored. Caution is required in clinical practice of the conclusion. Secondly, the included studies focused on the pain, OCR and drug requirement in the operating room, but did not involve the operation technique, postoperative visual rehabilitation and follow-up, which need further supplement in future research. Finally, the number of cases included in this study is 217, which is not a large sample size. When the sample size is further expanded, it is not known whether the conclusion of this paper will change.

Meta analysis will exaggerate the positive research results by excluding the gray literature, leading to decision-making errors. In order to ensure that the results of meta-analysis are more comprehensive and objective, and to overcome publication bias, it is recommended to include gray literature in meta-analysis. In this study, only published studies were included, so we re-searched several commonly used gray literature websites (GreyNet International: http://www.greynet.org/, Grey Literature Report: http://www.greylit.org/, Open Grey: https://opengrey.eu/), but no literature that meets the inclusion criteria of this paper was found. Therefore, the publication bias of this article is acceptable.

## Conclusion

Sub-Tenon’s bupivacaine injection can relieve short-term postoperative pain, reduce the incidence of OCR and vomiting, and reduce the use of drugs in strabismus surgery.

## Data Availability

The original contributions presented in the study are included in the article/**Supplementary Material**, further inquiries can be directed to the corresponding author.
